# Implementation of Orthogeriatrics in Portugal

**DOI:** 10.7759/cureus.35361

**Published:** 2023-02-23

**Authors:** Pedro Magalhães, Mariana Gonçalves, Fátima Silva, Tiago Fernandes, Agripino Oliveira, Rafaela Veríssimo

**Affiliations:** 1 Internal Medicine, Centro Hospitalar de Vila Nova de Gaia/Espinho, Vila Nova de Gaia, PRT

**Keywords:** orthogeriatrics, orthopaedics, internal medicine, hip fractures, frail elderly, geriatrics

## Abstract

Introduction: Orthogeriatrics is the subspecialty of geriatrics that is dedicated to the care of elderly patients with fragility fractures. The Orthogeriatrics Unit of the Vila Nova de Gaia Hospital Centre was the first unit created in Portugal in October 2015, in a co-management model.

Methods: Patients older than 65 years and with femur fractures were admitted to the unit after surgery. The department was run by internists with differentiation in geriatrics, and multidisciplinary support from orthopaedics, physiatrists, physiotherapists, nutritionists, and social workers, as well as rehabilitation nursing. A comprehensive multidisciplinary assessment was performed upon admission, including comprehensive geriatric assessment as well as postoperative monitoring of complications, investigation of fall mechanisms, functional rehabilitation, and outpatient orientation. Analysed variables included demographics, comorbidities, prior level of functionality, delay of orthopaedic surgery, complications, time of hospitalization, functional prognosis, and destination after discharge. Follow-up was maintained to assess short- and medium-term mortality. Kaplan-Meier curves and Cox regression were used for the statistical analysis of mortality.

Results: In four years of activity with 444 admissions, the typical patients were women (80.7%), with an average age of 84 years, coming from home (92%) after an accidental fall resulting in a proximal femur fracture. About half (54%) were previously autonomous, but with a high index of comorbidities (mean Charlson Index of 4.85), the most relevant of which were arterial hypertension (71%), malnutrition (46%), heart failure (35%), hyperlipidaemia (34%), osteoporosis (32%), and dementia (16%). During hospitalization, most patients had medical complications (86.3%), the most frequent ones being anaemia (45%), infections (35%), namely, urinary, respiratory, and surgical wound infections, acute heart failure (15%), and acute kidney injury (11%). Prevalent geriatric syndromes were also identified and corrected through protocols for delirium, urinary incontinence, pressure ulcers, and constipation. The mean length of stay was 12.49 days. At discharge, 75% presented a modified Rankin Scale score lower than 3 and 73% of patients were able to return home, with a low referral rate to long-term care facilities (5.9%). The in-hospital mortality rate was 2.65%. It was possible to maintain follow-up protocol after discharge in 343 patients, and the mortality at 12 months was 19.23% and at three years, it was 25.52%, with a risk of death almost doubled for patients discharged with a high degree of dependence (modified Rankin Scale score ≥ 3; OR: 2.19; p < 0.001).

Conclusion: We demonstrated reduced in-hospital mortality despite an elderly, frail population, with multiple previous comorbidities and a high number of inpatient intercurrences evidencing the importance of a good in-hospital co-management between internal medicine and orthopaedics, demonstrating the benefit of orthogeriatric units in patients with fragility fractures of the femur.

## Introduction

Orthogeriatrics is the subspecialty of geriatrics that is dedicated to the care of elderly patients with fragility fractures, providing pre-surgical optimization, management of perioperative complications, and rehabilitation.

The frailty of the elderly is reflected in the prognosis of femur fracture, with 10% of patients dying in the hospital within a month with the fracture accounting for less than half of all deaths [1]. Of those discharged, half regain their previous level of independence and half deteriorate [2].

Femoral neck fracture is a frequent occurrence in the elderly with multiple comorbidities and especially women, who are affected about twice as often as men. Its incidence has been increasing, as can be seen in a seven-year cohort (2005-2013) in Portugal, and occurring at an increasingly older age, demonstrating the contribution of population ageing in this trend [3]. Its mortality rate after discharge is high, with another Portuguese study reporting mortality rates of 10.2% at three months and 14.1% at six months and international studies reporting one-year mortality rates ranging from 15% to 36% [4-8].

To reduce the impact of this pathology, dedicated orthogeriatric units have emerged, demonstrating a decrease in hospitalization time as well as mortality at 30 days [9-12]. The internist is the most appropriate physician to follow the elderly patient with a femur fracture, particularly at critical moments of clinical decision [12].

The Orthogeriatrics Unit of the Vila Nova de Gaia Hospital Centre (CHVNGE) was the first of its kind created in Portugal, with a multidisciplinary team with co-management between internal medicine and orthopaedics [13,14]. The orthogeriatric unit objectives were to decrease in-hospital mortality as well as post-discharge medium and long-term survival and quality of life improvement, through several ways: early treatment of medical complications; prevention and treatment of geriatric syndromes such as frailty, functional limitation, falls, depression, polypharmacy, malnutrition, and cognitive impairment; reduction of pre-surgical waiting; recovery of the previous functional status in the shortest time and at the lowest possible cost; offering higher quality clinical care through daily clinical evaluation, managing complications, pre and post-surgical optimization; functional rehabilitation consultation; nutritional assessment; social assessment; and secondary prevention of bone fractures at discharge [9,15-22].

The aim of this study is to demonstrate that orthogeriatric units offer better care to older people with a femur fracture, lowering morbidity and mortality. Adequate planning for human resources, rehabilitation, and ward conditions during hospitalization and at discharge is essential and the authors aim to share this knowledge for the implementation of such new units.

## Materials and methods

The Orthogeriatrics Unit of our hospital centre began its activity in 2015. It included six beds in a geriatric medicine ward. The elderly population over 65 years of age compromised by a femur fracture were admitted to this unit after surgery. The service was run by internists with differentiation in geriatrics, with multidisciplinary support from orthopaedics, physiatrists, physiotherapists, nutritionists, and social workers, as well as rehabilitation nursing. A daily visit was made by internists, until clinical discharge home, nursing facility, or long-term care unit.

A multidisciplinary assessment was performed upon admission, including a comprehensive geriatric assessment of geriatric syndromes, postoperative monitoring of complications, investigation of the reason for the fall, and preparation of a functional rehabilitation plan and eventual outpatient orientation. A multidisciplinary team (internal medicine, orthopaedics, physiatry, rehabilitation nursing, social worker, and nutritionist) would meet once a week to discuss and address the patients' cases.

With the growth of the unit, additional patients were admitted, at times of greater demand, with occupancy of up to 10 beds in the last two years.

Analysed variables included age, gender, living situation, location and mechanism of fall, type of fracture, prior level of functionality, prior comorbidities, days from admission to surgery, complications during hospitalization, days from surgery to discharge, functionality, and destination at discharge. Follow-up was maintained to assess short- and medium-term mortality. Follow-up was maintained to assess short- and medium-term mortality. Kaplan-Meier curves and Cox regression were calculated for statistical and mortality analysis, using IBM SPSS Statistics, version 25 (IBM Corp., Armonk, NY).

## Results

During four years of activity (January 2016 to December 2019), 457 episodes corresponding to 444 patients were registered, with a re-admission rate of 2.92%. Characterization of the study population is summarized in Table 1. The population was predominantly female (80.7%). The median age was 84.50 years with 22.4% being over 90 years old. Most patients (92.1%) resided at home, and 14.4% of those who lived alone often presented with complex socioeconomic adversities, requiring an early and systematic approach by the team's social workers. The remaining patients lived in a nursing home (7.4%) and two were in government long-term care units.

**Table 1:** Characterization of the study population

Study population characterization	
Number of patients	444
Number of episodes	457
Age (median)	84.5
Female sex	358 (80.8%)
Living situation	
Home	409 (92.1%)
Nursing facility	33 (7.4%)
Government long-term care facility	2 (0.5%)
Assessment of previous functional independence	
Independent (Katz A or B)	239 (53.8%)
Dependent (Katz ≥ C)	205 (46.2%)
Location of fall	
At home	391 (88.1%)
Outside	53 (11.9%)
Mechanism of fall	
Positional instability	429 (96.6%)
Syncope	13 (2.9%)
External trauma	2 (0.5%)
Type of fracture	
Trochanteric fracture	263 (59.2%)
Femoral shaft fracture	138 (31.1%)
Sub-trochanteric fracture	39 (8.8%)
Intracapsular fracture	4 (0.9%)
Average time to surgery	2.7 days
Average time until discharge (mean)	12.49 days

Most of the falls occurred at home, because of sudden instability to change of position, stumbling, or slipping. The main diagnosis was proximal femur fracture, with most being transtrochanteric.

From the evaluation of the degree of previous autonomy through the Katz index [23], it was found that 54% of the population was classified as Katz A or B at admission, showing a high level of autonomy. From the functional assessment of gait, 85% had previously independent gait, and of those, 30% used gait aids (cane or crutches).

A high incidence of comorbidities was shown, with a mean Charlson index [24] of 4.85, corresponding to an estimated mean survival at 10 years of 26%. The most frequently observed comorbidities were hypertension (71%), heart failure (35%), hyperlipidaemia (34%), osteoporosis (32%), and dementia (16%). Only 2% of patients had no known previous comorbidities. The most frequent comorbidities and complications are summarized in Table 2.

**Table 2:** Most frequent comorbidities and complications during hospitalization

Comorbidities and complications during hospitalization	
Most frequent comorbidities	
Hypertension	315 (70.9%)
Heart failure	155 (34.9%)
Osteoporosis	142 (32%)
Dementia	71 (16%)
Most frequent complications	
Anaemia	206 (46.4%)
Acute heart failure	68 (14.9%)
Acute kidney injury	50 (11.3%)

With regard to nutritional assessment, malnutrition was frequently observed, with 22.4% of patients having a body mass index of less than 20.5 kg/m2. Almost half of the patients (46.3%), when applying the Nutritional Risk Screening (NRS) [25], had a score higher than 3, meaning a high risk of malnutrition. This was particularly evident in the most fragile individuals, defined by a score equal to or above 5 on the Rockwood Frailty Scale [26], with more than half (55.5%) being at high risk as opposed to 40% in the less fragile individuals.

Assessing the time to surgery, it was found that 62.2% were operated on within 48 hours with a median waiting time for surgery of 2.7 days. The most frequent reasons that led to delayed surgery were the absence of operating room availability, requiring reversal of anticoagulants, and clinical cardiorespiratory decompensation. After admission to the unit, the patients remained on average 12.49 days until discharge.

Concerning in-hospital evolution, only 13.7% of the patients had no complications. The main complications were anaemia (45.1%), infections (35%), namely, urinary, respiratory, and surgical wound infections, acute heart failure (14.9%), and acute kidney injury (11.3%). Prevalent geriatric syndromes were also identified and corrected through protocols for delirium, incontinence, pressure ulcers, and constipation.

Assessing functional status using the modified Rankin Scale [27] at discharge, 75% of patients achieved a score of less than 3, consistent with most patients (73%) being able to return home. A smaller group (5.9%) required a continued level of care that could not be provided at home, achieved by referral to government long-term rehabilitation facilities. Patients were also referred to orthopaedics, rehabilitation, and orthogeriatric outpatient consultation for follow-up.

**Figure 1:**
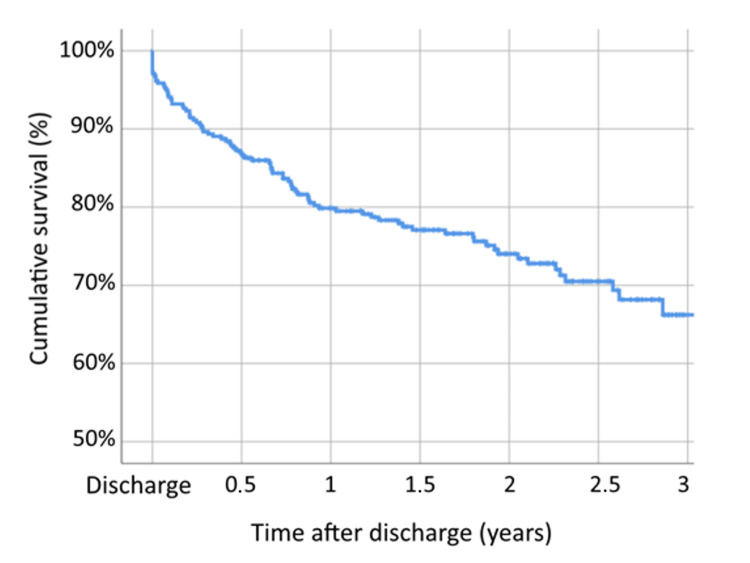
Kaplan-Meier survival curve during three years of follow-up

At the time of this study, follow-up information on mortality after discharge was possible in 343 patients, with an appropriate follow-up of 79.4%. The in-hospital mortality rate was 2.65%, it was 19.23% after 12 months, and at three years of follow-up, the mortality rate was 25.52% (Figure 1). The risk of death was almost doubled for patients discharged with a higher degree of disability, whether defined by a modified Rankin Scale score ≥ 3 (OR: 2.19; p < 0.001) or Katz index of A (OR: 1.93; p < 0.001).

## Discussion

With these differentiated units, better quality care for older people is provided and there is a reduced in-hospital mortality that is maintained in follow-up, even in the frail elderly population with multiple previous comorbidities. In this population, the mortality is lower when compared with traditional orthopaedics units.

Previously observed values in the literature reported up to 36% mortality at one year of follow-up [4-8]. A more direct comparison in a Portuguese study carried out in a similar population at an orthopaedic ward observed a mortality rate of 34.6% at one year after proximal femur fracture and, among these, 15.6% occurred during hospitalization, with more than a third (35.6%) occurring in the first three months after hospital discharge [28]. In contrast, our orthogeriatric unit observed mortality during hospitalization of 2.65%, at one year of 19.23%, and even at three years of follow-up of 25.52%.

These results should lead to reflection on the importance of specialized medical care adapted to this age group, which allows anticipating and avoiding intercurrences, adverse effects of medication, and the maintenance of the physical, functional, mental, and social state while focusing on the continuation of care for the family and the community. Most patients (73%) were able to recover or maintain their health status and autonomy, evidencing the importance of a specialized and multidisciplinary team.

Most comorbidities and complications were of medical, nonsurgical nature. This highlights the importance of creating specialized units with a multidisciplinary approach to femur fracture to reduce mortality. Scientific evidence has shown that in orthogeriatric units, patients recover faster and more effectively than in an orthopaedic hospitalization [9,12,16,29,30].

Also, there was a reduction in the number of complications during hospitalization, which is consistent with previous investigations [9,12,16], which improves rehabilitation much faster. This resulted in a relatively lower number of patients requiring long-term facility referral (5.9%) as well as lower degrees of disability.

Malnutrition was frequent, and systematic use of the NRS score revealed that almost half of our patients were at high risk for malnutrition, which often required adjustment of the dietary plan by the team nutritionist both in-hospital and at discharge.

A time to surgery of less than 48 hours is associated with a better prognosis [22]. This was not always possible and efforts must be made to avoid surgery delays, which in our hospital centre included increased operating room availability and surgical optimization such as protocols for anticoagulant reversal and systematic cardiorespiratory assessment [8].

Most patients were able to recover or maintain their health status, demonstrating a reduced in-hospital mortality that is maintained in follow-up despite an elderly, frail population, with multiple previous comorbidities and a high number of complications during hospitalization, evidencing the importance of a specialized and multidisciplinary team. This resulted in a relatively lower number of patients requiring long-term facility referral as well as lower degrees of disability.

Study limitations include a small sample and a loss of follow-up of 20.6%. Due to practical limitations on accessing follow-up records, post-discharge information was limited to mortality; therefore, causes of death and complications after discharge were unable to be considered. This might underestimate the complications of potentially early discharged patients.

## Conclusions

Co-management of the hospitalized patient, with particular attention to the orthogeriatric patient, is a benefit and its systemic implementation should be considered by hospital administrations. In Portugal, internal medicine, with differentiation in the area of geriatrics, is at the centre of decisions in the management of the orthogeriatric patient and is undoubtedly an asset in the treatment of these patients.

The experience acquired since the inception of this orthogeriatric unit indicates that a multidisciplinary approach is fundamental for the successful recovery and prevention of disability in the elderly. This team must include support not just from orthopaedics, but also physiatrists, physiotherapists, nutritionists, social workers, and rehabilitation nursing, all while focusing on community reintegration. This results in clear gains for patients, with reduced mortality and disability.
